# Diagnostic value of triple-phase bone scintigraphy for the diagnosis of infection around antibiotic-impregnated cement spacers

**DOI:** 10.1186/2193-1801-2-401

**Published:** 2013-08-27

**Authors:** Masahiko Ikeuchi, Yusuke Okanoue, Masashi Izumi, Goichi Fukuda, Koji Aso, Natsuki Sugimura, Teruhiko Kawakami, Toshikazu Tani

**Affiliations:** Department of Orthopaedic Surgery, Kochi Medical School, Kochi University, Kohasu, Oko-cho, Nankoku, Kochi, 783-8505 Japan; School of Health Science and Social Welfare, Kibi International University, Takahashi, Okayama, 720-0001 Japan

**Keywords:** Knee, Hip, Arthroplasty, Infection, Revision, Triple-phase bone scintigraphy

## Abstract

**Introduction:**

Two-stage revision arthroplasty is today considered as the gold standard for control of chronic deep prosthetic infection. Although the revision arthroplasty should only be considered when infection is eliminated, the diagnosis of residual infection prior to the revision remains a challenging problem.

**Materials and methods:**

We evaluated triple-phase bone scintigraphy as a useful diagnostic test for the detection of residual infection around the antibiotic-impregnated cement spacer in patients waiting for the second-stage revision hip or knee arthroplasty. Increased uptake of radioisotope in all three phases was considered positive for infection. The final diagnosis was based on histopathological results in addition to microbiologic examinations of surgical specimens.

**Results:**

Histopathological examination showed positive in 17 and negative in 13 out of 30 examinations. Among 17 samples positive for histopathology, there were only 4 samples positive for bacterial culture. All samples negative for histopathology showed negative for bacterial culture. The positive and negative predictive values of triple-phase bone scintigraphy for the presence of infection were 80% and 90%, respectively. The diagnostic sensitivity was 94% and the specificity was 69%.

**Conclusion:**

Triple-phase bone scintigraphy was useful in the diagnosis of infection around the articulating cement spacer. In particular, when triple-phase bone scintigraphy shows negative, the residual infection around the cement spacer is unlikely.

## Introduction

Periprosthetic infection occurs in less than 2% of patients (Phillips et al. 2006; Pedersen et al. 2010; Garvin and Konigsberg 2011) but nevertheless is one of the most devastating complications following hip and knee arthroplasties. Treatment options for periprothetic infection include irrigation and debridement (Van Kleunen et al. 2010), direct exchange arthroplasty (Ure et al. 1998; Silva et al. 2002), and two-stage revision arthroplasty (Biring et al. 2009; Borowski et al. 2012; Mahmud et al. 2012). Two-stage revision arthroplasty is today considered as the gold standard for control of chronic deep prosthetic infection (Cui et al. 2007; Sukeik and Haddad 2009; Mahmud et al. 2012). The procedure consists of implant removal, thorough debridement, placement of an antibiotic-impregnated cement spacer, a course of intravenous antibiotics, and a delayed second-stage revision arthroplasty (Cui et al. 2007).

The second-stage revision arthroplasty should only be considered when no signs of residual infection are present. Common investigations prior to the second-stage revision include serologic tests such as white blood cell count, erythrocyte sedimentation rate (ESR), and C-reactive protein (CRP), and joint fluid aspiration. However, normal serologic tests and negative cultures do not guarantee eradication of infection (Tunney et al. 1999; Bare et al. 2006; Bauer et al. 2006; Berbari et al. 2010). In particular, low-grade infection increases false-negative results (Sanzen and Sundberg 1997). Hence, the diagnosis of residual infection prior to the second-stage revision remains a challenging problem and requires additional diagnostic tests.

Triple-phase bone scintigraphy (TPBS) has been used to diagnose osteomyelitis (Schauwecker 1992) and periprosthetic infection (Rubello et al. 1995; Stumpe et al. 2004; Reinartz et al. 2005; Nagoya et al. 2008). When isotope uptake is increased in all three phases, periprosthetic infection can be differentiated from aseptic mechanical loosening with 80% accuracy for the hip and 81% accuracy for the knee (Stumpe et al. 2004; Nagoya et al. 2008; Reinartz 2009). The articulating cement spacer (Fehring et al. 2000; Biring et al. 2009) recently used during the waiting period is considered equivalent to a loose prosthetic component, once the postoperative acute phase is past. Therefore, it was expected that the TPBS would be useful in the diagnosis of infection around the articulating cement spacer. We have used the TPBS for this purpose since 2005. The aim of this retrospective study was to evaluate the TPBS as a useful diagnostic test for the detection of residual infection around the articulating cement spacer.

## Patients and methods

From January 2005 to December 2009, the TPBS followed by histopathological examination was performed 30 times in consecutive 15 patients who were treated for chronic deep periprosthetic infection at our institution. There were 4 men and 11 women, with a mean age at first operation 68 years (range 47-85). Infections occurred after primary total hip arthroplasty (THA) in 10 patients and after primary total knee arthroplasty (TKA) in 5 patients. Methicillin-resistant *Staphylococcus aureus* was detected in 5 patients, Methicillin-resistant *Staphylococcus epidermidis* in 2, *Staphylococcus aureus* in 3, Methicillin-resistant *Staphylococcus capitis* in 1, *Staphylococcus capitis* in 1, and undetected in 3. Three patients with undetected microorganism were diagnosed with periprosthetic infection by clinical symptoms and histological examinations of surgical specimens. Two patients with TKA had a sinus tract communicating with the loose prosthesis. In the other patient with THA, purulent fluid was repeatedly aspirated but no organism grew on culture. All patients had pain in the affected joint with elevation of serum CRP and synovial white blood cell count. Histopathological examination including frozen and permanent sections were positive (more than five polymorphonuclear leukocytes per high-power field (Tsaras et al. 2012)) in all patients.

First surgical procedure consisted of implant removal, irrigation, and meticulous debridement. At the end of the first procedure, articulating antibiotic-impregnated cement spacer was placed in all patients. After the first procedure, all patients were treated with intravenous antibiotics at least 6 weeks. The second-stage revision arthroplasty was considered when there were no signs of recurrent infections after cessation of antibiotic therapy. According to Greidanus et al. (Greidanus et al. 2007), ESR<22.5 mm/hr and CRP<1.35 mg/dl were considered negative for infection. In addition to these laboratory tests and joint aspirations, the TPBS was routinely evaluated before any surgical procedures including debridement, cement spacer exchange, and second-stage revision arthroplasty. The final decision to perform the second-stage revision arthroplasty was based on the results of frozen section in addition to intraoperative gross findings. A frozen section was considered positive for infection if there were more than five polymorphonuclear leukocytes per high-power field in at least five separate microscopic fields (Tsaras et al. 2012). If the results of intraoperative frozen section were positive or purulent fluid was observed during surgery, thorough debridement and cement spacer exchange were repeated. Paraffin-embedded permanent sections were prepared following every surgery. A permanent section was considered positive for infection if there were more than five polymorphonuclear leukocytes per high-power field in at least five separate microscopic fields. The average number of surgical procedures required until the second-stage revision arthroplasty was 2.4 times (1-4 times) and the average treatment period was 4.8 months (3-13 months). Patients were advised to use wheelchair during the intervals between the first procedure and the revision arthroplasty. The mean follow-up after the revision arthroplasty was 3.2 years (range 2-7 years). To be judged infection-free at follow-up, a patient had to be free of clinical signs for infection (fever, local pain, redness, warmth, sinus tract infection) and have a normal CRP level and no radiological sign of osteolysis (Fink et al. 2009).

The TPBS was performed at a mean of 12 weeks (8-20 weeks) after the previous operation. The bone scintigraphy images were obtained after injecting technetium-99m-labelled diphosphonate (555mBq) and using GCA-7200 (Toshiba Medical Systems Corporation, Tochigi, Japan). The TPBS consisted of three phases (Nagoya et al. 2008); the blood flow phase, reflecting hyperaemia, was immediately after infusion of the tracer; the blood pool phase, reflecting inflammation, was between 3 and 5 minutes later, and the late phase, reflecting bone activity, between 3 and 4 hours after infusion. The TPBS images were transferred to a workstation (RS 6000, Intelli Station E Pro, IBM, USA) equipped with a 1,024 × 768 pixel solution monitor (IBM Co., Tokyo, Japan). A square region of interest of 5×5 pixels was placed over the most intense focus. Although scintigraphy has poor spatial resolution, it was possible to localize major anatomical structures. The accumulation of isotope within the region of interest in question was quantified using CIS-image (IBM Co., Tokyo, Japan). According to Nagoya et al. (Nagoya et al. 2008), the accumulation in the hip was expressed as a percentage compared with the gradation in the contralateral femoral artery for the blood flow phase, the femoral vein for the blood pool phase, and the anterior iliac crest for the late phase. Increased uptake of the isotope in the blood flow and blood pool phases was noted when the density was ≧75% of the gradation in the femoral artery and femoral vein, respectively. Increased uptake in the late phases was noted when the density was equivalent to, or greater than, 90% of gradation in the anterior iliac crest. Similarly, the accumulation in the knee was compared with the gradation in the contralateral popliteal artery for the blood flow phase, the popliteal vein for the blood pool phase, and the tibial tuberosity for the late phase. Increased uptake in all three phases of TPBS was considered positive for infection (Nagoya et al. 2008). For assessing the intra- and inter-observer agreement of measurements, two observers performed the measurements twice, one week apart.

### Statistical analysis

Histopathological results in addition to microbiologic examinations of surgical specimens represented the reference standard in all patients. Although the final decision to perform the second-stage revision arthroplasty was based on the frozen section results, the final histopathological diagnoses were made using permanent sections. Sensitivity, specificity, positive predictive value and negative predictive value for the TPBS in the diagnosis of infection were calculated. Intra- and inter-observer agreements were assessed using the interclass correlation coefficients (ICC). All analyses were performed by using a statistical software program (SPSS, version 11.5; SPSS, Chicago, IL).

## Results

There was no recurrence of infection after the revision arthroplasty except 2 patients. Recurrent infection was noted 1 year after the revision THA in one patient and 2 years after the revision TKA in another patient. The patient with infected revision THA underwent delayed reimplantation surgery again. Another patient with infected revision TKA underwent arthrodesis of the knee because of soft tissue problems. There was no recurrence of infection in these two patients (2 and 3 year follow-up respectively).

**Figure 1:**
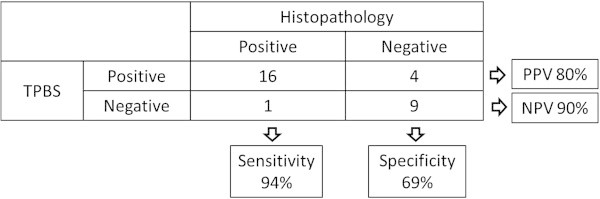
**Relationship between the results of triple-phase bone scintigraphy and histopathological examination.** TPBS: Triple-phase bone scintigraphy, PPV: positive predictive value, NPV: negative predictive value.

**Figure 2:**
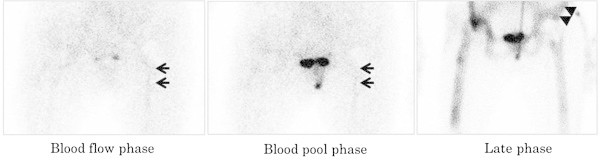
**Triple-phase bone scintigraphy in a patient without infection around the cement spacer of the hip.** Arrows indicate the contralateral femoral artery in the blood flow phase and the femoral vein in the blood pool phase. Arrowheads indicate the contralateral anterior iliac spine.

**Figure 3:**
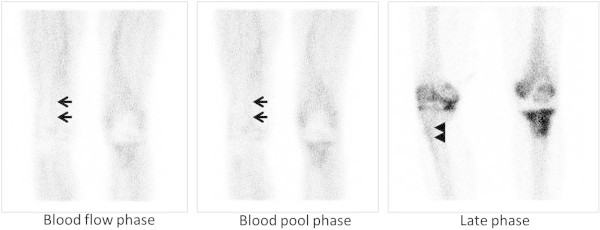
**Triple-phase bone scintigraphy in a patient with residual infection around the cement spacer of the knee.** Arrows indicate the contralateral popliteal artery in the blood flow phase and the contralateral vein in the blood pool phase. Arrowheads indicate the contralateral tibial tuberosity.

There was no discordance between the results of frozen and permanent sections. Histopathological examination showed positive in 17 and negative in 13 out of 30 examinations. Among 17 samples positive for histopathology, there were only 4 samples positive for bacterial culture. All samples negative for histopathology showed negative for bacterial culture. Figure 1 shows the relationship between the results of TPBS and histopathological examination. The positive and negative predictive values for the presence of infection were 80% and 90%, respectively. The diagnostic sensitivity was 94% and the specificity was 69%. Figure 2 shows a patient without infection around the cement spacer of the right hip. The late phase was positive, while the blood flow and blood pool phases were negative. Figure 3 shows a patient with residual infection around the anitibiotic-impregnated cement spacer of the left knee, which was confirmed by intraoperative frozen section. The TPBS was positive in all three phases.

The inter-observer ICC was 0.82 and the intra-observer ICC was 0.88, indicating good intra- and inter-observer agreement on the TPBS measurements.

## Discussion

There is a need to establish additional diagnostic tests for residual infection prior to the second-stage revision. To the best of our knowledge, this is the first study to evaluate the diagnostic value of TPBS for the diagnosis of infection around antibiotic-impregnated cement spacers. Based on our results, the diagnostic sensitivity of TPBS was 94% and the specificity was 69%. Although the specificity was lower than the previous report on infected hip prosthesis (Nagoya et al. 2008), the high negative predictive value (90%) makes the TPBS a clinically useful test. When the TPBS are negative, the residual infection around the cement spacer is unlikely. This information is particularly useful in patients with underlying inflammatory disease or malignancy, because CRP and ESR levels often fluctuate. In contrast, positive TPBS requires meticulous examinations including repeated aspirations and new laboratory markers such as interleukin-6, procalcitonin, and TNF-alpha (Bottner et al. 2007).

Infection around the cement spacer induces inflammatory responses such as neovascularization and granulation formation. This condition is equivalent to septic loosening of prosthesis, which can be diagnosed using TPBS. Therefore, TPBS was useful in detecting residual infection around cement spacers. However, the first two phases of TPBS reflecting hyperemia and inflammation are not necessarily related to infection. It was expected that main factor affecting the results is postoperative period. In this study, TPBS was performed at least 8 weeks after the previous surgery. Although postoperative acute inflammation is considered to attenuate within the period of time, it is possible that longer intervals between previous surgery and TPBS could reduce false-positive cases.

Recently, FDG-PET has been successfully used to detect periprosthetic infection (Zhuang et al. 2001; Delank et al. 2006; Zoccali et al. 2009; Mayer-Wagner et al. 2010). It is reported that FDG-PET is a more accurate diagnostic test than TPBS (Reinartz et al. 2005; Reinartz 2009). A systematic review reported that accuracy of FDG-PET was 89% for hip and 83% for knee arthroplasty, while TPBS was 80% for hip and 81% for knee (Reinartz 2009). In addition, Huang et al. (Huang et al. 2011) recently reported that FDG-PET was a feasible tool to help in detecting infection around antibiotic-loaded cement spacers. Although PET is highly effective procedure for detecting infection around prosthesis and cement spacers, its limitations are the restricted availability and the costs. TPBS yields a slightly lower accuracy, but excels in simplicity and cost-effectiveness (Reinartz 2009). Because FDG-PET is not yet common, we believe that TPBS still has an important role in diagnosis of residual infection around prosthesis and cement spacers.

There are several limitations in this study. Firstly, this is a retrospective study with a relatively small number of patients during a 5-year period. Larger prospective studies are needed to corroborate these findings. Secondly, histopathological examination was used as the reference standard for the diagnosis of infection. At the final follow-up, recurrent infection was noted in two patients despite negative results in histopathology. Although infection probably occurred long after the revision surgery in these two patients, we cannot exclude the possibility of the presence of infection at the revision surgery. Lastly, hips and knees were simultaneously evaluated in this study. Some authors reported that accuracy of FDG-PET is lower in knee than in hip prosthesis (Zhuang et al. 2001; Reinartz 2009). Although it is reported that TPBS is less affected by the complexity of the knee joint (Reinartz 2009), the results might differ between hips and knees. We did not compare TPBS results between hips and knees because the number of patients with infected knees was quite small.

In conclusion, TPBS was useful in the diagnosis of infection around the articulating cement spacer. In particular, the high negative predictive value makes the TPBS a clinically useful test. When the TPBS are negative, the residual infection around the cement spacer is unlikely. We believe that TPBS is particularly useful in patients with underlying inflammatory disease or malignancy, because serological examinations such as CRP and ESR are not always reliable in the diagnosis of residual infection.

No benefits in any form have been received or will be received from a commercial party related directly or indirectly to the subject of this article.

## Consent

Written informed consent was obtained from the patient for the publication of this report and any accompanying images.
